# Structural and Functional Diversity of Acidic Scorpion Potassium Channel Toxins

**DOI:** 10.1371/journal.pone.0035154

**Published:** 2012-04-12

**Authors:** Zong-Yun Chen, Dan-Yun Zeng, You-Tian Hu, Ya-Wen He, Na Pan, Jiu-Ping Ding, Zhi-Jian Cao, Mai-Li Liu, Wen-Xin Li, Hong Yi, Ling Jiang, Ying-Liang Wu

**Affiliations:** 1 Wuhan Center for Magnetic Resonance, State Key Laboratory of Magnetic Resonance and Atomic and Molecular Physics, Wuhan Institute of Physics and Mathematics, Chinese Academy of Sciences, Wuhan, People's Republic of China; 2 State Key Laboratory of Virology, College of Life Sciences, Wuhan University, Wuhan, People's Republic of China; 3 Key Laboratory of Molecular Biophysics, Ministry of Education,College of Life Science and Technology, Huazhong University of Science and Technology, Wuhan, Hubei, People's Republic of China; Virginia Commonwealth University, United States of America

## Abstract

**Background:**

Although the basic scorpion K^+^ channel toxins (KTxs) are well-known pharmacological tools and potential drug candidates, characterization the acidic KTxs still has the great significance for their potential selectivity towards different K^+^ channel subtypes. Unfortunately, research on the acidic KTxs has been ignored for several years and progressed slowly.

**Principal Findings:**

Here, we describe the identification of nine new acidic KTxs by cDNA cloning and bioinformatic analyses. Seven of these toxins belong to three new α-KTx subfamilies (α-KTx28, α-KTx29, and α-KTx30), and two are new members of the known κ-KTx2 subfamily. ImKTx104 containing three disulfide bridges, the first member of the α-KTx28 subfamily, has a low sequence homology with other known KTxs, and its NMR structure suggests ImKTx104 adopts a modified cystine-stabilized α-helix-loop-β-sheet (CS-α/β) fold motif that has no apparent α-helixs and β-sheets, but still stabilized by three disulfide bridges. These newly described acidic KTxs exhibit differential pharmacological effects on potassium channels. Acidic scorpion toxin ImKTx104 was the first peptide inhibitor found to affect KCNQ1 channel, which is insensitive to the basic KTxs and is strongly associated with human cardiac abnormalities. ImKTx104 selectively inhibited KCNQ1 channel with a K_d_ of 11.69 µM, but was less effective against the basic KTxs-sensitive potassium channels. In addition to the ImKTx104 toxin, HeTx204 peptide, containing a cystine-stabilized α-helix-loop-helix (CS-α/α) fold scaffold motif, blocked both Kv1.3 and KCNQ1 channels. StKTx23 toxin, with a cystine-stabilized α-helix-loop-β-sheet (CS-α/β) fold motif, could inhibit Kv1.3 channel, but not the KCNQ1 channel.

**Conclusions/Significance:**

These findings characterize the structural and functional diversity of acidic KTxs, and could accelerate the development and clinical use of acidic KTxs as pharmacological tools and potential drugs.

## Introduction

Potassium channels include a large family of membrane proteins that are important in numerous cellular functions including maintenance of resting membrane potential, control of cardiac and neuronal excitability, release of neurotransmitters, muscle contractility, and hormone secretion [1]–[3]. Potassium channels therefore provide unique targets for the development of new drugs to treat cancer, autoimmune diseases, and neurological and cardiovascular disorders [4].

Over 400 million years, the scorpion has evolved a vast array of toxins in different species. In the last 30 years, these toxins have been used to probe the structural and functional relationships of potassium channels and to develop channel-selective pharmacological tools and drug candidates [5]–[7]. Scorpion toxins that act on potassium channels (KTxs) are divided into four groups: α-, β-, γ-, and κ-KTxs [8], [9]. Structurally, four different scaffold motifs within the scorpion toxins have been identified: CS-α/β [10], CS-α/α [11], [12], ICK (inhibitor cystine knot) [13], and DDH (disulfide-directed β-hairpin) [14]. Sequence analyses have shown that most KTxs are well-known basic toxins with pIs >7. Structure and function studies have further demonstrated that these basic KTxs modulate Kv1.x channels [5], [15], Kv11.1 channels [16], [17], large-conductance Ca^2+^-activated K^+^ channels (BKCa) [18], intermediate-conductance Ca^2+^-activated K^+^ channels (IKCa) [19], [20], and small conductance Ca^2+^-activated K^+^ channels (SKCa) [21], [22]. Among a large of potassium channels, most are still shortage of peptide blockers, and discovery of novel KTxs targeting these channels remains a huge challenge [23], [24].

In this study, we characterized a group of acidic KTxs with pIs <7 isolated from different scorpion species and identified seven new acidic KTxs belonging to three new subfamilies of α-KTxs, and two new members of the κ-KTx2 subfamily. In addition to the known pharmacological effects of acidic KTxs on Kv1.x and SKCa channels [6], [25], our electrophysiological studies demonstrated for the first time that the α-KTx ImKTx104 blocked the KCNQ1 channel with a K_d_ of 11.69 µM. ImKTx104 was found to have a modified cystine-stabilized α-helix-loop-β-sheet (CS-α/β) fold. In addition to ImKTx104 toxin, other acidic KTxs exhibit different pharmacological effects on potassium channels. HeTx204 peptide, with a CS-α/α fold scaffold, blocks both Kv1.3 and KCNQ1 channels. StKTx23 toxin, with a CS-α/β fold motif, inhibits Kv1.3 channels, but not the KCNQ1 channel. Together, these findings demonstrated the structural and functional diversity of acidic KTxs, which could be used to mine novel KTxs affecting KCNQ1 and other potassium channels without the specific blockers.

## Materials and Methods

### Construction and screening of cDNA libraries

The construction of cDNA libraries from *Lychas mucronatus*, *Isometrus*, *maculates*, *Scorpiops tibetanus*, *Scorpiops jendeki*, and *Heterometrus spinifer* venom glands has been described previously [26], [27]. Clones carrying an insert of 300–1,000 base pairs potentially encoding venom peptide precursors were selected for DNA sequencing. Nucleotide sequences used in this study have been deposited in the GenBank database (http://www.ncbi.nlm.nih.gov) under accession numbers EU252205 (ImKTx104), EU163848 (LmKTx2), EU163849 (LmKTx51), FD664509 (StKTx23), FD664203 (HeTx203), FD664204 (HeTx204), GT028769 (LmKTx95), GH548288 (SjKTx32), and GH548307 (SjKTx51).

### Bioinformatic analyses of acidic toxins

A search of the GenBank database by BLASTP (BLAST 2.2.23) was used to find potential homologues of the new acidic peptides. Toxin sequences were aligned by Clustalx 1.83 (http://www.ebi.ac.uk) and phylogenetic trees were constructed. Three-dimensional (3D) structures of all newly identified toxins were obtained by comparative modeling using SWISS-MODEL, a fully automated protein structure homology-modeling server (http://swissmodel.expasy.org/), followed by energy minimization using AMBER 9 [28].

### Purification and characterization of acidic peptides

Acidic peptides were synthesized and characterized as previously described [5], [29]. To uniformly ^15^N-label ImKTx104, bacteria were cultured in M9 Minimal Media containing ^15^NH_4_Cl as the sole nitrogen source. Expression was induced by the addition of 1 mM isopropyl β-D-1-thiogalactopyranoside. After harvesting the cells by centrifugation, the fusion protein was purified as previously described [30]. After cleavage, ^15^N-labeled ImKTx104 was further purified by high pressure liquid chromatography (HPLC). Eluted fractions containing the isolated ImKTx104 protein were lyophilized and stored at −20°C until use in nuclear magnetic resonance (NMR) experiments.

### Electrophysiological studies of acidic KTxs

The cDNAs encoding Kv1.1, Kv1.2, Kv1.3, SKCa and BKCa were cloned into the pIRES2-EGFP vector (Clontech, Mountain View, CA, USA) for co-expression with green fluorescent protein as previously described [31]. The cDNAs encoding TRPV1, KCNQ1 and mink subunit were cloned into the pcDNA3.1 vector (Invitrogen, Carlsbad, CA, USA). All vectors containing the entire coding channel sequences were transiently transfected into HEK293 cells (China Center for Type Culture Collection, Wuhan, China) using Sofast™ Transfection Reagent (Sunma Biotech, Xiamen, China), and channel currents were measured 1 to 3 days after transfection. Current measurements and data acquisition were obtained with an EPC 10 patch clamp amplifier (HEKA Elektronik, Lambrecht, Germany), which was controlled by PULSE software (HEKA Elektronik) as previously described [8]. We evaluated the effect of ImKTx104 on KCNQ1 channel with the equilibrium dissociation constant (K_d_), an equivalent method with IC_50_. K_d_ was calculated using the equation: K_d_ = [ImKTx104]/((τ_off_/τ_on_)−1), where the τ_on_ means the time constant of binding process, and τ_off_ means the time constant of washing off process.

### NMR spectroscopy

The purified sample contained ∼2 mM ^15^N-labeled ImKTx104 in 20 mM phosphate buffer, pH 5, dissolved in 90% H_2_O/10% D_2_O (vol/vol). NMR measurements were made at 27°C using a Bruker AVANCE-III 800 spectrometer. All chemical shifts were externally referenced to the methyl resonance of sodium 2,2-dimethyl-silapentane-5-sulfonate (0 ppm). Data were collected in the phase-sensitive detection mode using standard pulse sequences. WATERGATE-W5 was employed for solvent suppression at the end of Total Correlation Spectroscopy (TOCSY) and Nuclear Overhauser Effect Spectroscopy (NOESY) [32]. Flip-back pulse was used for water suppression in ^15^N, ^1^H-Heteronuclear Single Quantum Coherence (HSQC) and ^15^N-edited 3D NOESY-HSQC [33]. The ^1^H-^15^N heteronuclear NOE experiments were acquired using ^1^H-^15^N HSQC based sequence with (NOE experiment) and without (NONOE experiment) the ^1^H saturation before the start of the experiment. The saturation of ^1^H was obtained by applying 120° ^1^H pulses spaced by 5 ms. In NONOE experiment, a relaxation delay of 2 s was employed, which is prior to the proton presaturation period of 3.5 s applied in NOE experiment. H-D exchange rates were also investigated by acquiring a series of ^1^H-^15^N HSQC spectra for 6 hours at 27°C immediately after the sample was dissolved in D_2_O with the identical concentration and pH as before. Each HSQC experiment took half an hour [34].

### NMR data processing and analysis

NMR data were processed using NMRPipe and were analyzed with the XEASY module of the Cara program [35]. Proton resonance assignments were determined from TOCSY (mixing times of 30 ms and 70 ms), NOESY (mixing times of 120 ms, 200 ms, and 300 ms), and ^15^N-edited 3D NOESY-HSQC (mixing time of 120 ms).

Distance constraints of ImKTx104 were defined primarily from NOESY spectrum at a mixing time of 120 ms using the CALIBA program, and also from H-D exchange experimental results. Backbone dihedral angle constraints were derived from proton chemical shifts using TALOS [36]. A family of 200 structures was calculated using the Cyana 2.1 program [37], starting from randomly generated conformers in 10,000 annealing steps. Twenty conformers with minimal target functions were selected to undergo energy minimization using the AMBER force field to represent the structure of ImKTx104 in a liquid environment. The quality of each calculated structure was evaluated using PROCHECK-NMR [38]. Figures and graphical analyses of structures were created using MOLMOL [39]. Structure coordinates were deposited into the Protein Data Bank with the code 2LIX. The ^1^H–^15^N steady-state NOE values were given by the ratio of the signal intensities of an identical peak obtained from the NOE and NONOE spectrum, respectively. The measurement error was estimated from the signal-to-noise ratio of the corresponding peak.

## Results

### Molecular diversity of acidic KTxs

**Figure 1:**
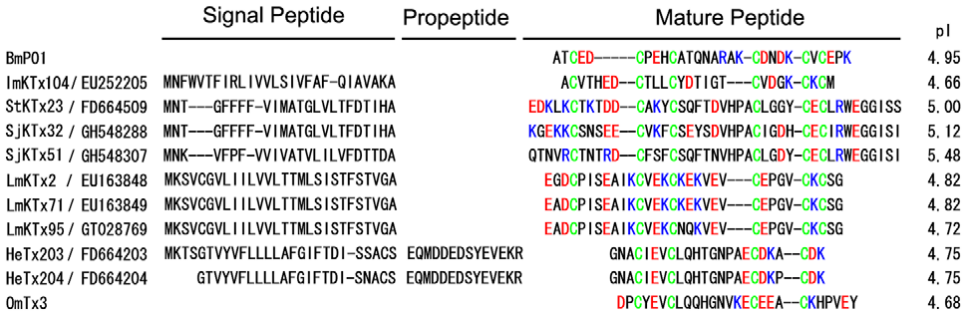
Sequence alignment of nine new KTxs. Cysteines are shadowed in green. Acidic and basic residues are shown in red and blue, respectively. Isoelectric points (pIs) were calculated using compute pI/Mw tool (http://web.expasy.org/compute_pi/).

In this work, we describe the isolation and identification of novel acidic KTxs, and identify nine new transcripts encoding precursors of acidic KTxs from the cDNA libraries of *Lychas mucronatus*, *Isometrus*, *maculates*, *Scorpiops tibetanus*, *Scorpiops jendeki*, and *Heterometrus spinifer* venom glands. According to their sequences and structural features, we have named these new acidic KTxs ImKTx104, LmKTx2, LmKTx71, LmKTx95, StKTx23, SjKTx32, SjKTx51, HeTx203, and HeTx204. Fig. 1 shows that all of the new acidic KTx precursors contain a typical signal peptide, as predicted by SignalP 3.0 (http://www.cbs.dtu.dk/services/SignalP/). The mature peptides are composed of 24 to 42 highly variable amino acids. All toxins, except HeTx203 and HeTx204, contain six cysteines with alignment patterns similar to the known acidic toxin α-KTxs BmP01 [25], [40], indicating that they may adopt the CS-α/β fold scaffold motif. The HeTx203 and HeTx204 peptides may adopt the CS-α/α fold scaffold using four cysteines, a pattern similar to that of the known acidic κ-KTx OmTx3 [12]. All new KTxs are acidic peptides with pI values 4.66 to 5.48. Analyses of sequence alignment and structural features showed that seven of the nine KTxs belonged to three new α-KTx subfamilies (α-KTx28: ImKTx104; α-KTx29: StKTx23, SjKTx32, and SjKTx51; α-KTx30: LmKTx2, LmKTX71, and LmKTx95) and two were new members of the κ-KTx subfamily (κ-KTx2: HeTx203 and HeTx204). These data demonstrate the significant molecular diversity of acidic KTxs.

#### New subfamily α-KTx28 with acidic ImKTx104

**Figure 2:**
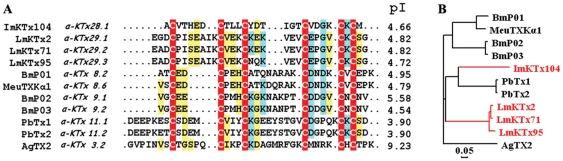
New subfamilies of acidic α-KTx28 and α-KTx29. (A) Multiple sequence alignment of ImKTx104, LmKTx2, LmKTx71, and LmKTx95 with other α-KTxs; (B) Phylogenetic tree of four new acidic toxins with other known acidic α-KTxs.

Compared with known acidic and basic KTxs, ImKTx104 shares low sequence similarity but has similar disulfide bridges (Fig. 2). In contrast to classical basic KTxs, ImKTx104 likely lacks both the crucial pore-blocking Lys27 (AgTX2 numbering) and the additional hydrophobic residue (Phe or Tyr) as the functional dyad [41]. ImKTx104 has four negatively charged residues (Glu6, Asp7, Asp14, and Asp21) and only two positively charged residues (Lys23 and Lys25), making ImKTx104 a much stronger acidic peptide, with a pI value of 4.66. According to widely accepted nomenclature [9], [30], ImKTx104 is the first member of a new subfamily, α-KTx28.

#### New subfamily α-KTx29 with acidic LmKTx2, LmKTx71 and LmKTx95

As shown in Fig. 2, the three new acidic toxins LmKTx2, LmKTX71, and LmKTx95 show extensive sequence homology with only one or two residue difference. However, they have low sequence homology with other known acidic and basic KTxs. LmKTx2, LmKTX71, and LmKTx95 have similar pI values of ∼4.82 due to their almost identical sequences. Similar to the classical acidic α-KTxs, six cysteine residues retain the conserved disulfide bridge pattern, suggesting that these three acidic toxins belong to a new α-KTx subfamily, designated α-KTx29.

#### New subfamily α-KTx30 with acidic StKTx23, SjKTx32 and SjKTx51

**Figure 3:**
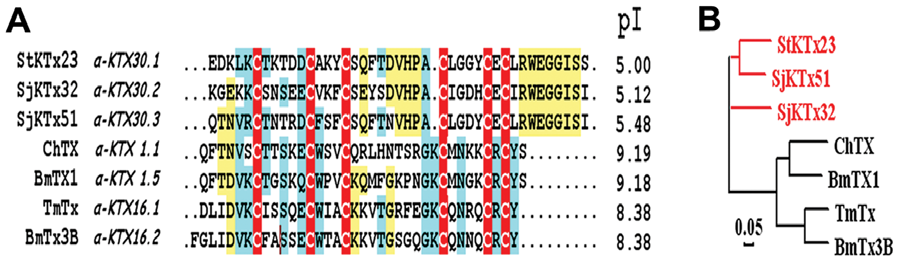
New subfamily of acidic α-KTx30. (A) Multiple sequence alignment of StKTx23, SjKTx32, and SjKTx51 with other α-KTxs; (B) Phylogenetic tree of three new acidic toxins with other related α-KTxs.

As shown in Fig. 3A, three of the newly identified acidic peptides, StKTx23, SjKTx32, and SjKTx51, have the longest sequence (42 residues) among known acidic KTxs, demonstrating extensive sequence heterogeneity among the acidic KTxs. Although there is high sequence homology in the C-terminal regions of StKTx23, SjKTx32, and SjKTx51, there is variability when comparing their N-terminal regions. These three newly identified toxins have differing numbers of charged residues and subsequent differences in pI values, ranging from 5.00 for StKTx23 to 5.48 for SjKTx51. Due to their simialr disulfide bridge patterns with the classical scorpion toxin ChTX, these three peptides may adopt classical CSα/β fold although they belong to a new group, the α-KTx30 subfamily (Fig. 3B).

#### New members of the κ-KTx2 subfamily with acidic HeTx203 and HeTx204

**Figure 4:**
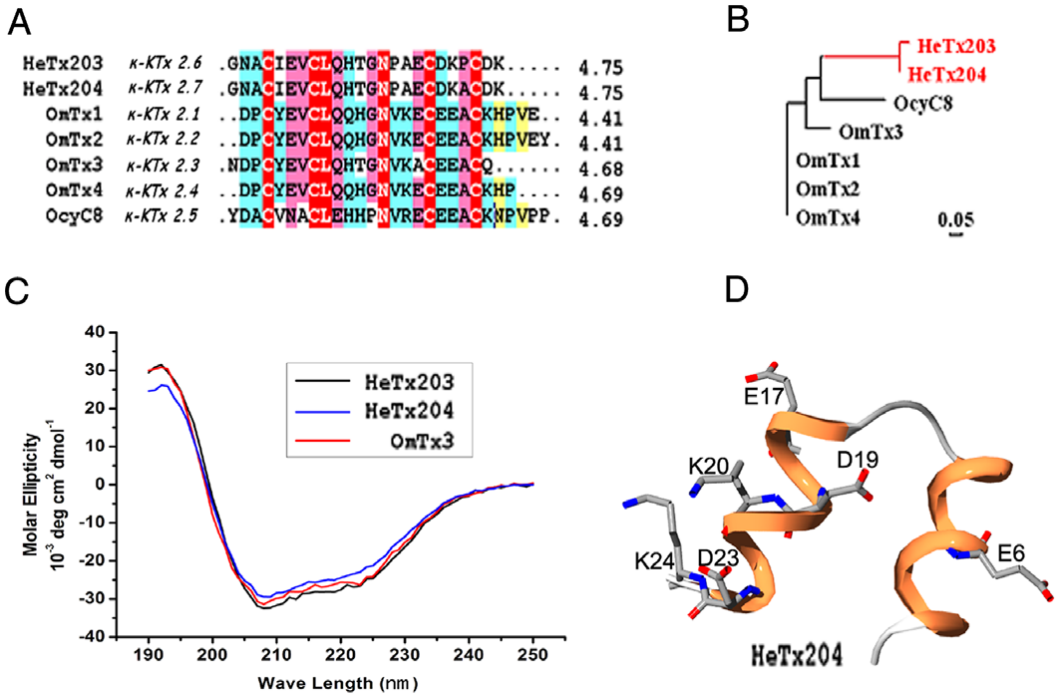
Two new members of the kappa-KTx2 subfamily. (A) Multiple sequence alignment of HeTx203 (kappa-KTx2.6) and HeTx204 (kappa-KTx2.7) with other kappa-KTx2 members; (B) Phylogenetic tree of two new kappa-KTx2 toxins with other members; (C) Circular dichroism spectra of HeTx203, HeTx204, and OmTx3; (D) Model structure of HeTx204 with OmTx3 as a template.

The κ-KTx2 subfamily is composed of five highly homologous members, including OmTx1-4 and OcyC8 [12], [42]. Our results demonstrated that HeTx203 and HeTx204 differ by only a single residue and show extensive homology with the κ-KTx2 subfamily members (Fig. 4A and B). The two conserved disulfide bridges in HeTx203 and HeTx204 could adopt the known CS-α/α fold scaffold motif consistent with their CD spectra and their corresponding modeled structures, which confirmed two α-helical regions linked by a short loop (Fig. 4C and D). The charged residues are mainly distributed in the second helical domain near the C-terminal region.

### Novel pharmacological effects of acidic KTxs

**Figure 5:**
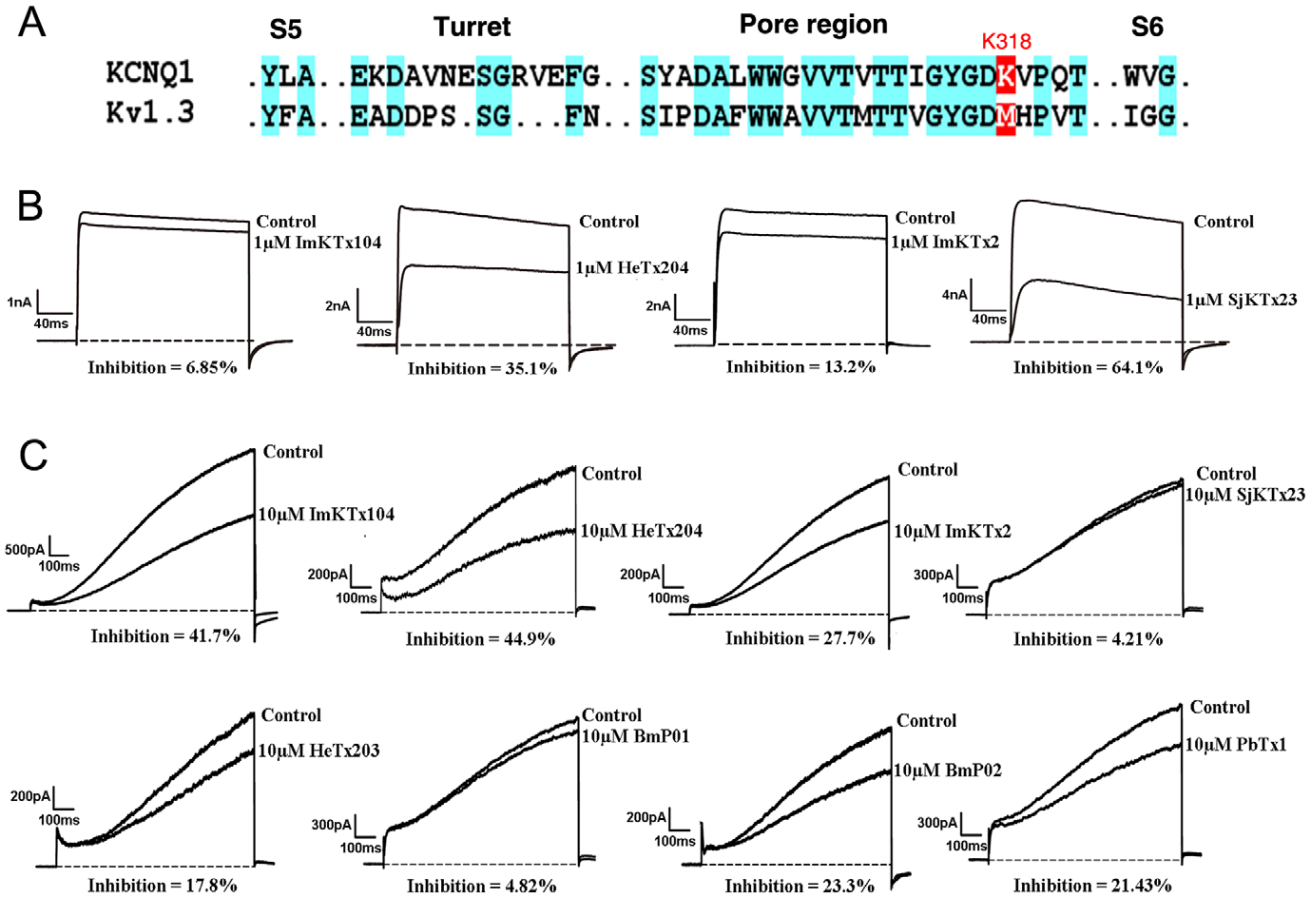
Functional evaluation of representative acidic KTxs. (A) Sequence alignment of Kv1.3 and KCNQ1 channels; (B) Inhibition of Kv1.3 channel current with 1 µM ImKTx104, HeTx204, LmKTx2, and StKTx23; (C) Inhibition of KCNQ1 channel current with representative acidic KTxs (10 µM). Average inhibition of different toxins is indicated (n>3).

Previous studies indicated that acidic KTxs selectively blocked Kv1.3 channels with low affinities (micromolar range). One exception is the toxin MeuTXKα1, which specifically inhibits the Kv1.3 channel with an IC_50_ value of 2.36±0.90 nM [6], [12], [43]. We therefore investigated the effects of the newly identified acidic KTxs on the Kv1.3 channel. Acidic ImKTx104, LmKTx2, StKTx23, HeTx204, HeTx203, BmP01, BmP02, and PbTx1 were expressed and purified as previously described [5], [30], and as demonstrated for ImKTx104 in Fig. S1. Representative toxins from the four subfamilies had different effects on the Kv1.3 channel (Fig. 5B). Toxins ImKTx104 and LmKTx2 had a minor effect on the Kv1.3 channel, whereas SjKTx23 and HeTx204 effectively blocked the Kv1.3 channel when each was tested at micromolar levels.

For further characterizing the pharmacological features of acidic KTxs, we transferred our vision from the classical basic toxin-sensitive potassium channels to KCNQ1 channel, which is still lacking peptide inhibitor. As shown in Fig. 5A, KCNQ1 contains Lys318 adjacent to the conserved channel selectivity filter “GYGD” motif. Substitution of Lys318 changes the binding affinity of basic KTxs from insensitivity to high affinity (nanomolar) by eliminating the strong electrostatic repulsion between the basic KTxs and the channel [44]. The acidic KTxs could compensate for the repulsion of basic KTxs because negatively charged residues could interact with Lys318 in the KCNQ1 channel pore region. As predicted, we showed that acidic KTxs effectively inhibited the KCNQ1 channel current.

To demonstrate the pharmacological effects of acidic KTxs on the KCNQ1 channel, XE991 was first used to confirm the KCNQ1 channel current (Fig. S2). As shown in Fig. 5C, each acidic toxin had different effects on the KCNQ1 channel. Toxins ImKTx104 and HeTx204 effectively blocked the KCNQ1 channel at 10 µM concentration, while LmKTx2 and HeTx203 only weakly inhibited the KCNQ1 channel at the same concentration, and 10 µM StKTx23 did not inhibit KCNQ1 channels. In comparison, the previously studied acidic BmP01, BmP02, and PbTx1 [25], [43] were only weak inhibitors of KCNQ1 channels, with BmP01 having the lowest inhibitory activity at 10 µM.

When measured at 10 µM, ImKTx104 blocked the KCNQ1 channel current with τ_on_ = 81.32 s and τ_off_ = 11.73 s, giving a K_d_ of 11.69 µM (Fig. S2). The HeTx204 toxin could not be removed, and therefore, the τ_off_ value could not be measured. These results further demonstrate the novel pharmacological properties of acidic KTxs on the KCNQ1 channel, which is insensitive to classical basic KTxs.

### Selectivity of toxins ImKTx104 and HeTx204 towards KCNQ1 channel

**Figure 6:**
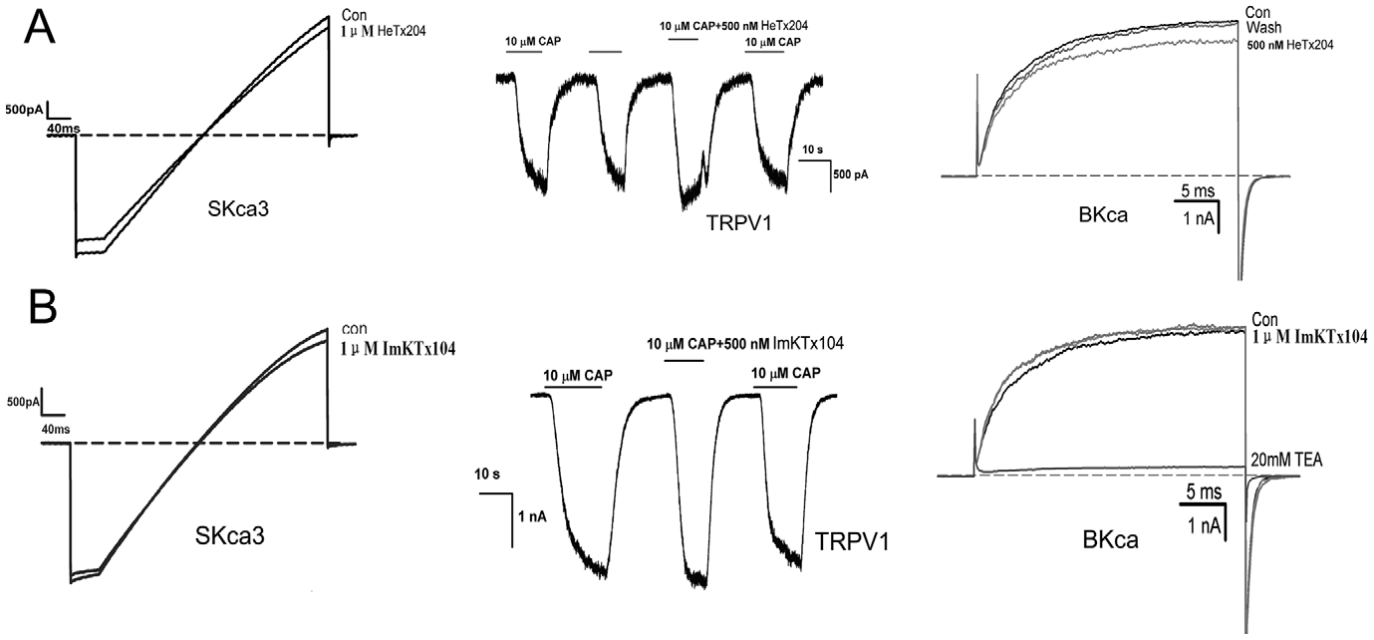
Selectivity of toxins ImKTx104 and HeTx204. (A) Inhibition of SKCa, TRPV1, and BKCa channel currents with 1 µM HeTx204; (B) Inhibition of SKCa, TRPV1, and BKCa channel currents with 1 µM ImKTx104. Representative results are shown (n>3).

The acidic peptide BmP01 weakly inhibits the SKCa channel [25]. Based on the pharmacological properties of acidic ImKTx104 and HeTx204 on Kv1.3 and KCNQ1 channels, we further investigated their effects on different types of potassium channels. As shown in Fig. 6, toxins ImKTx104 and HeTx204 both had negligible effects on BKCa, SKCa, and TRPV1 channels at 1 µM concentration. In addition, ImKTx104 had no effect on Kv1.1 and Kv1.2 channels (Fig. S2). Our pharmacological data therefore showed that acidic HeTx204 blocked both Kv1.3 and KCNQ1 channels, whereas ImKTx104 was the first selective peptide inhibitor for the KCNQ1 channel.

### Unique structural scaffold of acidic ImKTx104

Toxin ImKTx104 has low sequence homology to known acidic and basic KTxs (Figs. 1 and 2) and showed novel inhibition of the KCNQ1 channel (Figs. 5 and 6). Although ImKTx104 has six cysteine residues and has the classical scaffold CXXXC—GXC—CXC in its primary structure, it was not clear whether this toxin adopts the classical CS-α/β fold scaffold in its 3-D spatial structure. This was determined using our previously described NMR technique [31].

**Figure 7:**
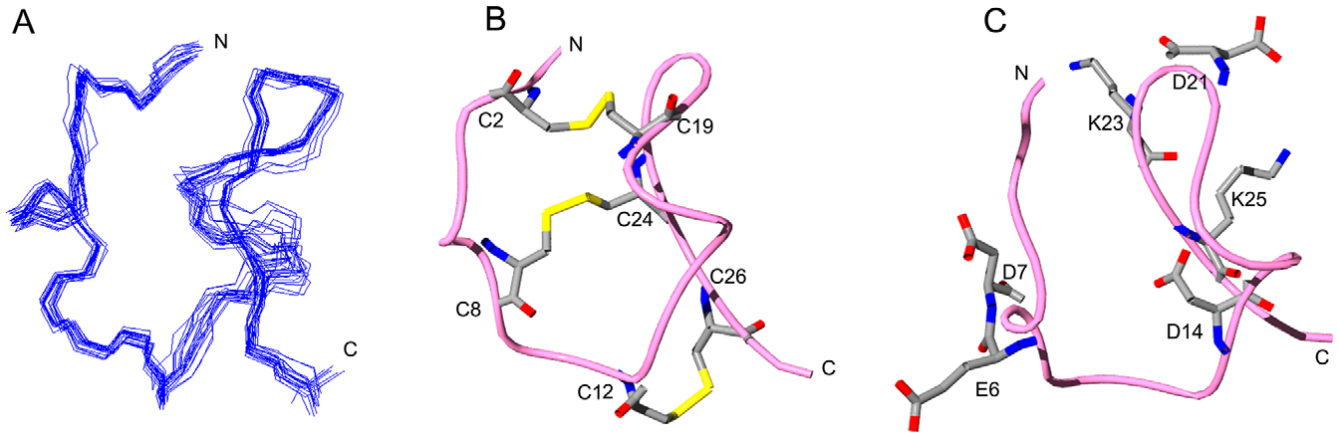
Structure of ImKTx104 by ^1^H, ^15^N-NMR. (A) Superposition of the backbone atoms of the best 20 structures, fitted to residues 1 to 27; (B) Ribbon representation of the structure closest to the mean with the disulfide bridges shown; (C) Ribbon representation of the structure with the acidic and basic residues shown.

A total of 296 non-redundant upper limit constraints (Table S1) from the assigned NOE cross-peaks were included in the structure calculations, together with 18 TALOS torsion angle constraints (Figs. S3 and S4). Based on the position of cysteines from initial structural calculations, six disulfide bond constraints, paired in C^2^-C^19^, C^8^-C^24^, and C^12^-C^26^ were added to the input of NOE upper limit distance constraints. Hydrogen bond constraints of CO_20_HN_23_ were also added in the calculation based on the results of H-D exchange experiment. From the initial 200 structures, 20 structures corresponding to those exhibiting the lowest target functions were selected. None of these structures exhibit distance deviations greater than 0.5 Å or dihedral angle deviations greater than 4° (Fig. 7A). A ribbon representation of the “most typical” (closest-to-the-mean) conformer is shown in Fig. 7B and Fig. 7C. The disulfide bridge pattern of toxin ImKTx104 was similar to that of the classical α-KTxs, such as ChTX and BmP01 [45], [46]. However, ImKTx104 has no apparent α-helix and β-sheet structures. Only a 3_10_-helix remains from residue E^6^ to C^8^, and the backbone trend of the fragment from T^18^ to K^25^ looks like a hairpin motif. As for the spatial distribution of charged residues, two basic residues Lys23 and Lys25 were adjacent to Asp14 and Asp21 and far from Asp6 and Asp7 (Fig. 7C). Asp14 was buried, and perhaps inaccessible, within the ImKTx104 toxin. This is unusual for KTxs with short sequence length. In whole, the structure of ImKTx104 is still stabilized by three conserved disulfide bridges that are consistent with its primary structure, but ImKTx104 has no apparent secondary structures except a 3_10_-helices. Different from the classical CSα/β fold that comprises one or two short α-helices connected to a triple-stranded antiparallel β-sheet stabilized by three or four disulfide bonds [40], [45], [47], ImKTx104 adopts a modified CSα/β fold, which indicates the diverse scaffold of small disulfide-rich scorpion toxins with common cysteine framework [48].

## Discussion

**Figure 8:**
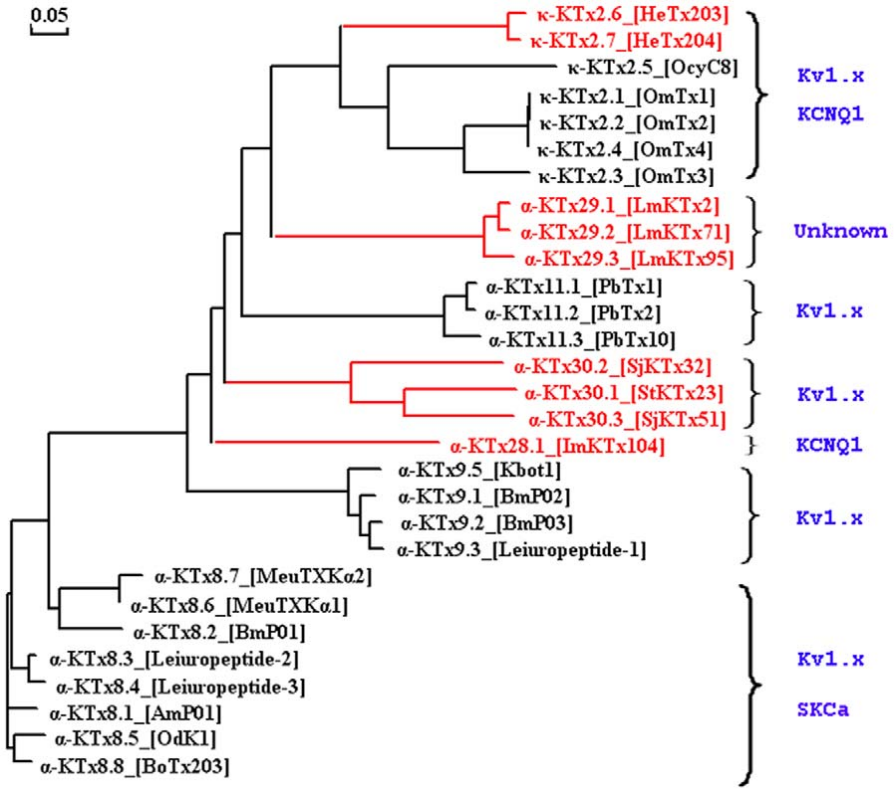
Molecular diversity of acidic KTxs. Sequence alignment of nine new KTxs with all known acidic KTxs. The nine new acidic KTxs are marked in red and their possible targets are shown.

During the last 30 years, basic KTxs have been widely used to probe the structure and function of potassium channels, including Kv1.x, Kv11.1, BKCa, IKCa, and SKCa [17], [18], [25]. However, novel KTx inhibitors are needed both for channel structure-function studies and as potential drugs to treat potassium channel-related disorders for which there are no inhibitors [4]. During the past 10 years, about 20 acidic KTxs have been isolated from scorpion toxins; however, the pharmacological function of most acidic toxins remains unknown [49]. Here, we report the identification of nine new acidic KTxs, including members of three new α-KTx subfamilies and two new members of the known κ-KTx2 subfamily. Toxin ImKTx104 was the first identified member of the new α-KTx28 subfamily. It has low sequence homology to known acidic and basic KTxs (Fig. 2). Toxins LmKTx2, LmKTX71, and LmKTx95, which share high sequence homology, are members of the new α-KTx29 subfamily (Fig. 2). Toxins StKTx23, SjKTx32, and SjKTx51 are three members of a new α-KTx30 subfamily. Comprised of 42 residues, they are the longest in sequence length among the known acidic KTxs (Fig. 3). Toxins HeTx203 and HeTx204 are two new members of the κ-KTx2 subfamily, and differ from each other in only a single amino acid residue (Fig. 4). Because the phylogenetic tree of known acidic KTxs shows great molecular diversity (Fig. 8), it may be possible to find additional acidic KTxs using transcriptomic and peptidomic strategies [27], [50]

Kv1.x channels are often used to identify acidic KTxs with potential pharmacologic functions. Acidic toxin MeuTXKα1 was the first selective Kv1.3 inhibitor with high affinity (nanomolar range) [6]. Many acidic toxins block the Kv1.x channel with low activity (micromolar range), such as BmP01, PbTx1, OmTx1, and OmTx3 [12], [43]. We report that representative toxins of four subfamilies have different effects on the Kv1.3 channel. Similar to known functions of acidic KTxs, toxins StKtx23 and HeTx204 effectively blocked Kv1.3 channels at micromolar concentrations, whereas toxins ImKTx104 and LmKTx2 showed less effect. These data suggest that individual acidic KTxs have different effects on Kv1.x channels.

To further investigate the pharmacological features of acidic KTxs, we selected the KCNQ1 channel as a target for the following reasons: (I) It is closely linked to cardiac abnormalities and has no previously identified peptide inhibitor [1]; (II) Lys318 adjacent to the conserved channel selectivity filter motif “GYGD” confers insensitivity of basic KTxs towards the KCNQ1 channel [44]. In basic KTx-sensitive channels, there are no basic residues near the selective filter (Fig. 5A); (III) Selective inhibitors are potential drugs [4]. After screening five newly identified acidic peptides and three known peptides (BmP01, BmP02, and PbTx1), we found that each of the acidic toxins had different effects on KCNQ1: toxin ImKTx104 was the first selective inhibitor of KCNQ1 with a K_d_ of 11.69 µM; toxin HeTx204 blocked Kv1.3 and KCNQ1 at micromolar concentration; toxins LmKTx2, HeTx203, BmP02, and PbTx1 could weakly inhibit KCNQ1 at 10 µM level; and toxins StKTx23 and BmP01 had a minor effect on KCNQ1 at 10 µM concentration. Because toxin BmP01 acts on the SKCa channel [25], the observation that acidic KTxs block KCNQ1 demonstrates the functional diversity of acidic KTxs and suggests that ImKTx104 could provide a template for the design of improved toxins that are specific to KCNQ1 channels.

**Figure 9:**
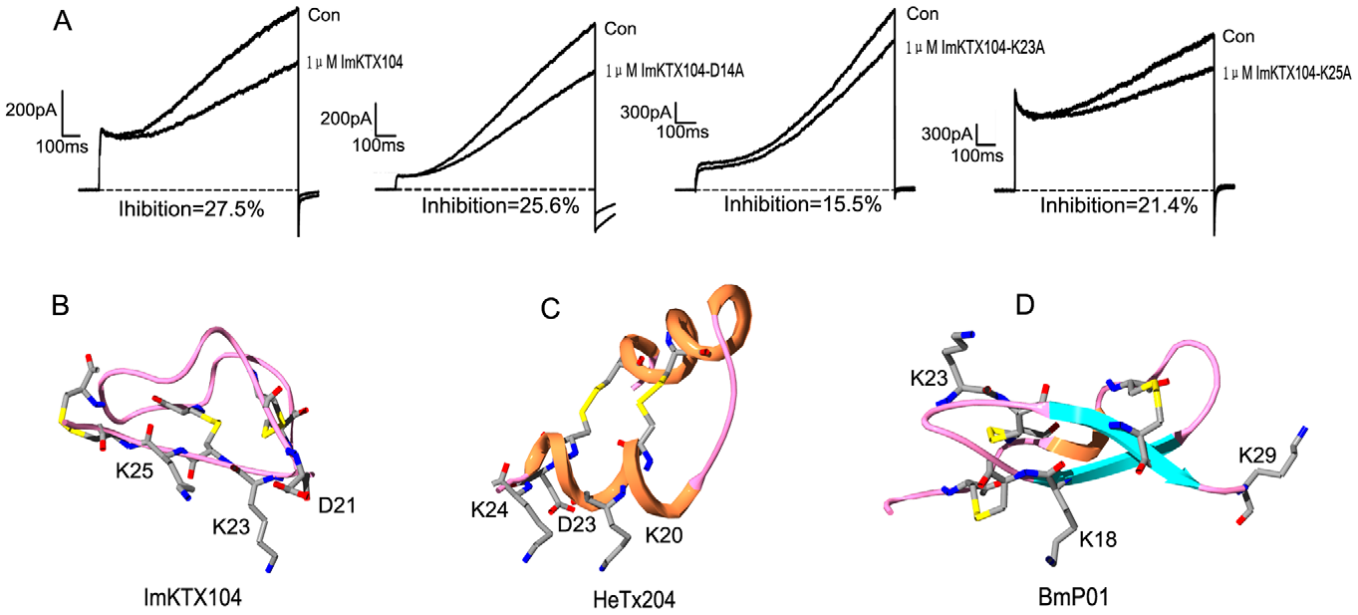
Structure-function relationships of acidic KTxs. (A) The effect of ImKTx104 mutants on KCNQ1 channel current, and Average inhibition is indicated (n>3); (B–D) Possible binding interfaces of ImKTx104, HeTx204, and BmP01, respectively.

Structurally, only two CS-α/β and CS-α/α folds were seen in acidic α-KTxs with two or three disulfide bridges (Fig. 9) [12], [40]. We determined the structure of ImKTx104, an acidic toxin with much lower sequence homology with other known acidic toxins (Fig. 2) that exhibits a novel pharmacological function towards KCNQ1 (Figs. 5 and 6). In contrast to the classical CS-α/β fold that comprises one or two short α-helices connected to a triple-stranded antiparallel β-sheet stabilized by three or four disulfide bonds [8], [47], ImKTx104 adopted a random coil structure except a short 3_10_ helix. Then, we investigated the backbone dynamics of ImKTx104 by ^1^H–^15^N steady-state NOE measurements and compared its structure with that of BmP01. BmP01, an acidic scorpion toxin with a classical CSα/β fold, has a globular structure consisting of a helix from C^3^ to T^12^ connected by a turn to a two stranded antiparallel β-sheet from A^15^ to D^20^ and K^23^ to E^27^ (Fig. S6A) [40]. Instead of the long helical region, ImKTx104 has a short 3_10_-helix from E^6^ to C^8^ (Fig. S6C), but the V^3^-L^10^ fragment of ImKTx104 remains rigid and the NOE values of these residues are around 0.7 (Fig. S5). The structural rigidity also presents in the region of T^18^-C^26^ that the trend of backbone is comparable to the β-sheet segment of A^15^ to E^27^ of BmP01, but no regular antiparallel β-sheet NOE patterns were found in NMR spectra of ImKTx104. H-D exchange experiments show that no slowly exchangeable amide protons are observed except a strong peak of HN_23_ and two weak signals of HN_20_ and HN_15_, (Fig. S6B). The hydrogen bond CO_20_HN_23_ and a relative slow exchangeable HN_20_ stabilize the V^20^-K^23^ turn, and make the NOEs of V^20^, D^21^ and K^23^ reach 0.67. These analyses indicate that ImKTx104 lacks the classical secondary structure elements, however, it still presents the conservation of globular structure characteristics of the α-KTx toxins [40].

Between the helix and β-sheet fragments of BmP01, there is a short loop from Q^13^ to N^14^. However, in the corresponding region of ImKTx104, there is a much longer loop from L^11^ to G^17^ that may result into shortage of the apparent β-sheet in ImKTx104 structure (Fig. S6). The NOE value sharply drops to the lowest point of 0.52 at C^12^, followed by a gradual increase from Y^13^ to T^15^. It reaches 0.64 at T^15^ due to the relative slow exchangeable amide proton HN_15_ which is close to CO_25_. The NOE value of I^16^ decreases again and the HN_17_ peak in HSQC and NOESY spectra is not found (Fig. S5). The T^15^-G^17^ fragment looks more likely an additional loop extruding out of the structure of ImKTx104, but the observations of inter-residue NOE cross peaks, such as γH and βH of I^16^ to HN_19_, and βH of T^15^ to the methyl proton of V^20^, confirm the existence of this loop region. These results suggest that toxin ImKTx104 has a modified CSα/β fold, in which the classical α-helices and β-sheets were replaced by the random coils but the whole conformation is still stabilized by three conserved disulfide bridges. It would be a new elaboration of the classical CSα/β scaffold, thus revealed the structural versatility of small disulfide-rich scorpion toxins [48].

To further study the structure-function relationship of ImKTx104, Asp14, Lys23, and Lys25 were each substituted by an alanine residue. As shown in Fig. 9A, Lys23 was the most crucial to ImKTx104 activity because its replacement had the greatest effect on KCNQ1 channels. Both Asp14 and Lys25 have weak effects on ImKTx104 activity. These data indicate that ImKTx104 recognition of the KCNQ1 channel involves basic residues on the toxin. Lys23 presumably blocks the channel pore, while the adjacent Asp21 could interact with the Lys318 near the channel selectivity filter, thus changing the affinity of ImKTx104 towards KCNQ1. The acidic BmP01 peptide has the classical CS-α/β fold structure, with a conserved “AKC-19” motif existing among the basic KTxs. The main functional residues might be the pore-blocking Lys18 and the two basic residues, Lys23 and Lys29 on BmP01 that inhibit the Kv1.3 channel at micromolar concentration [6]. HeTx204, with its CS-α/α fold motif, has two basic residues, Lys20 and Lys24, that might be responsible for inhibitory activity towards the Kv1.3 and KCNQ1 channels. These representative motifs further verify the structural and functional diversity of the acidic KTxs.

In conclusion, we have identified nine novel acidic KTxs that can be grouped into three new α-KTx subfamilies and new members of the known κ-KTx2 subfamily. Of these, the ImKTx104 toxin was found to have a unique structural fold without apparent α helix and β-sheet , which was different from the classical CS-α/β fold present in the acidic α-KTxs. Functionally, different acidic toxins showed differential pharmacological effects on Kv1.3 and KCNQ1 channels. Acidic ImKTx104 is a selective KCNQ1 channel inhibitor, whereas HeTx204 can block both Kv1.3 and KCNQ1 channels. These novel findings not only reveal the structural and functional diversity of acidic KTxs but could also lay the basis for development of acidic KTxs as specific pharmacological tools and potential drugs.

## Supporting Information

Figure S1 Expression, purification, and characterization of peptide ImKTx104. (A) Tricine-SDS-PAGE analysis of expression of GST-ImKTx104 fusion protein and purification of rImKTx104. Lane 1, molecular mass markers; lane 2, purified GST fusion protein after affinity chromatography and concentration; lane 3, cleaved fusion protein by Enterokinase; lane 4, purified rImKTx104 by HPLC; (B) HPLC profile of the fusion protein cleaved by Enterokinase and mass spectrum of rImKTx104; The measured value of rImKTx104 by MALDI-TOF-MS is 2919.9 Da, and the calculated value is 2919.5 Da.
(TIF)

Figure S2 (A) Pharmacological identification of KCNQ1 (mink) channel by XE991; (B) K_d_ value of ImKTx104 towards KCNQ1 channel; (C) and (D) Inhibition of Kv1.1 and Kv1.2 channel currents with 1 µM ImKTx104. Representative results are shown (n>3).
(TIF)

Figure S3 NMR spectra of ImKTx104 at pH 5, 27°C. (A) ^15^N, ^1^H-HSQC. (B) Expanded Hα/HN and Hβ/HN region of NOESY spectrum at a mixing time of 120 ms.
(TIF)

Figure S4 Summary of the NOE connectivities according to NOESY spectrum at 120 ms of mixing time and CαH chemical shift index (CSI). Bar thickness indicates the intensity of NOE connectivities, with thicker bars representing stronger NOEs.
(TIF)

Figure S5 Values of backbone amide heteronuclear ^1^H–^15^N NOEs of ImKTx104. The NOE values are shown by red spot and line by residue number, and black bars indicate standard deviations.
(TIF)

Figure S6 Structure comparison of ImKTx104 with the classical CSα/β scorpion toxin BmP01. (A) Structure of BmP01 (PDB code:1WM7). The representative amino acid residues were marked. (B) and (C), Structure of ImKTx104 (PDB code:2LIX). The representative amino acid residues were also marked.
(TIF)

Table S1 Structure statistics for ImKTx104 at pH 5 (20 structures).
(TIF)
