# Supplementary figures and images for: Structural and Functional Diversity of Acidic Scorpion Potassium Channel Toxins
Source: PLoS One. 2012 Apr 12;7(4):e35154. doi: 10.1371/journal.pone.0035154 (PMC3325286; doi:10.1371/journal.pone.0035154)

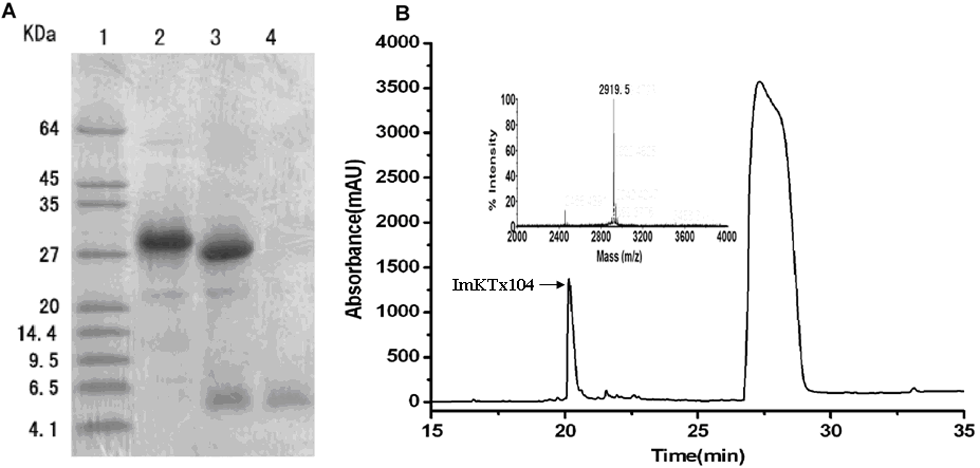

Supplement: Figure S1 — Expression, purification, and characterization of peptide ImKTx104. (A) Tricine-SDS-PAGE analysis of expression of GST-ImKTx104 fusion protein and purification of rImKTx104. Lane 1, molecular mass markers; lane 2, purified GST fusion protein after affinity chromatography and concentration; lane 3, cleaved fusion protein by Enterokinase; lane 4, purified rImKTx104 by HPLC; (B) HPLC profile of the fusion protein cleaved by Enterokinase and mass spectrum of rImKTx104; The measured value of rImKTx104 by MALDI-TOF-MS is 2919.9 Da, and the calculated value is 2919.5 Da. (TIF) [file pone.0035154.s001.tif]

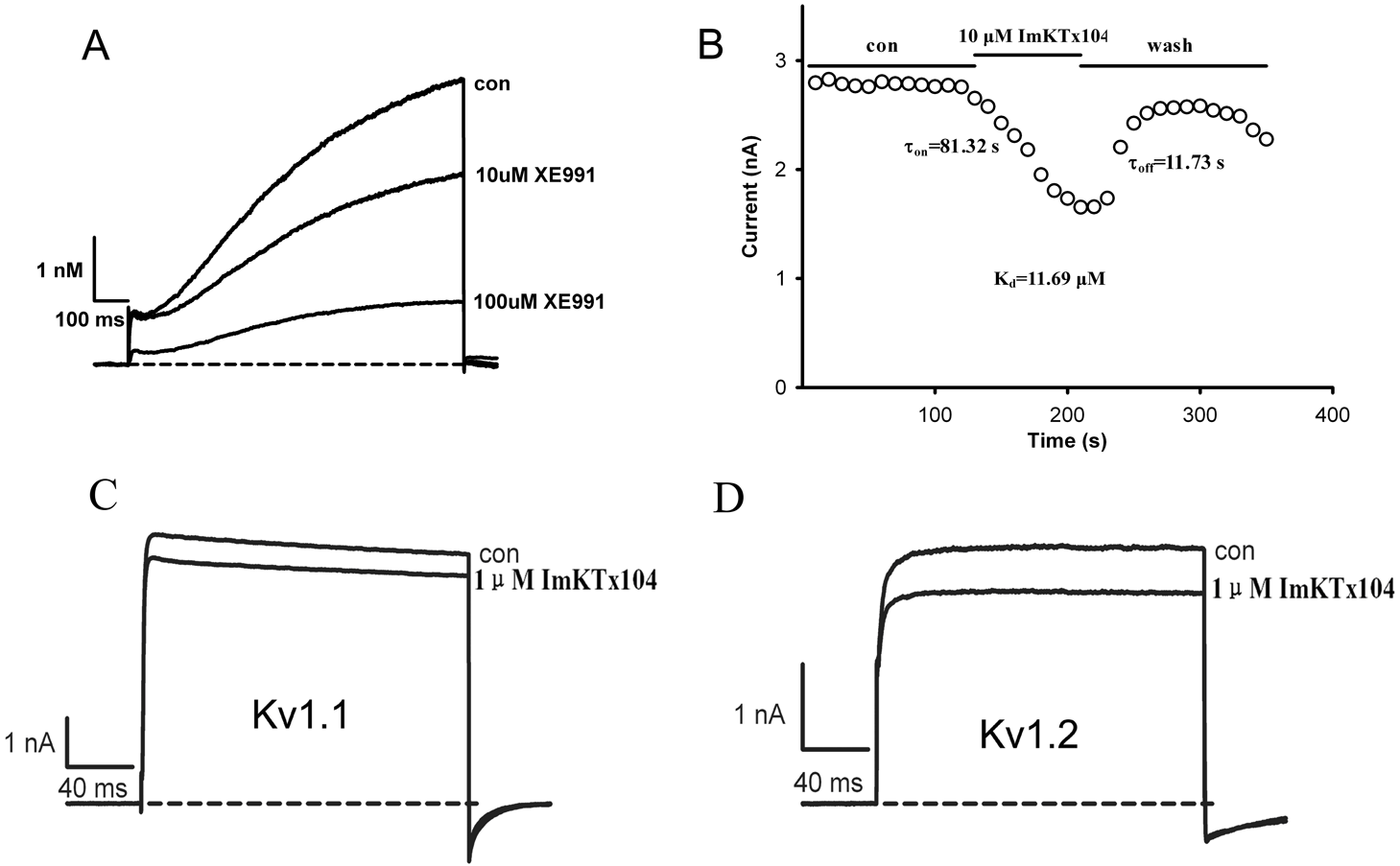

Supplement: Figure S2 — (A) Pharmacological identification of KCNQ1 (mink) channel by XE991; (B) Kd value of ImKTx104 towards KCNQ1 channel; (C) and (D) Inhibition of Kv1.1 and Kv1.2 channel currents with 1 µM ImKTx104. Representative results are shown (n>3). (TIF) [file pone.0035154.s002.tif]

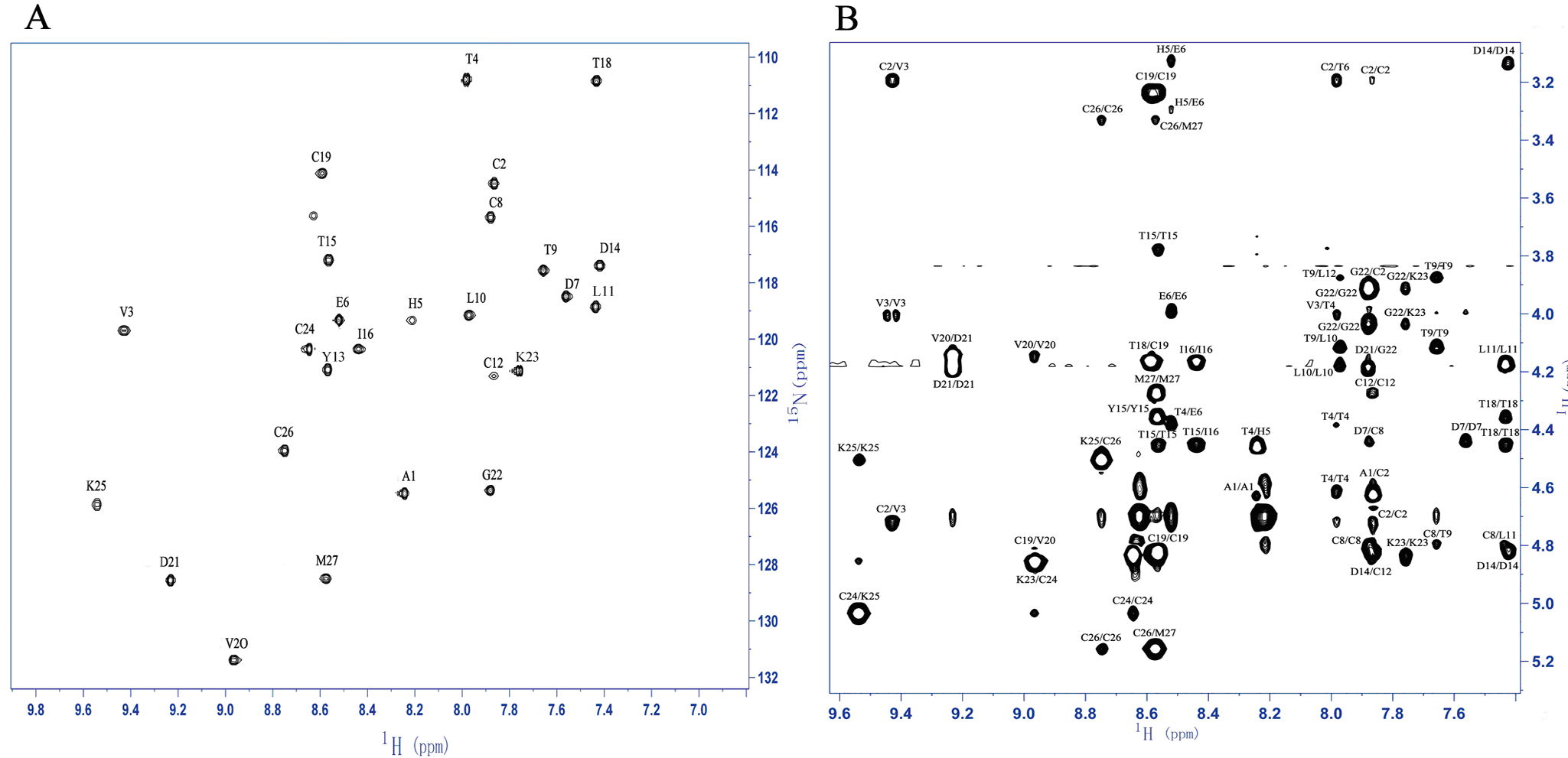

Supplement: Figure S3 — NMR spectra of ImKTx104 at pH 5, 27°C. (A) 15N, 1H-HSQC. (B) Expanded Hα/HN and Hβ/HN region of NOESY spectrum at a mixing time of 120 ms. (TIF) [file pone.0035154.s003.tif]

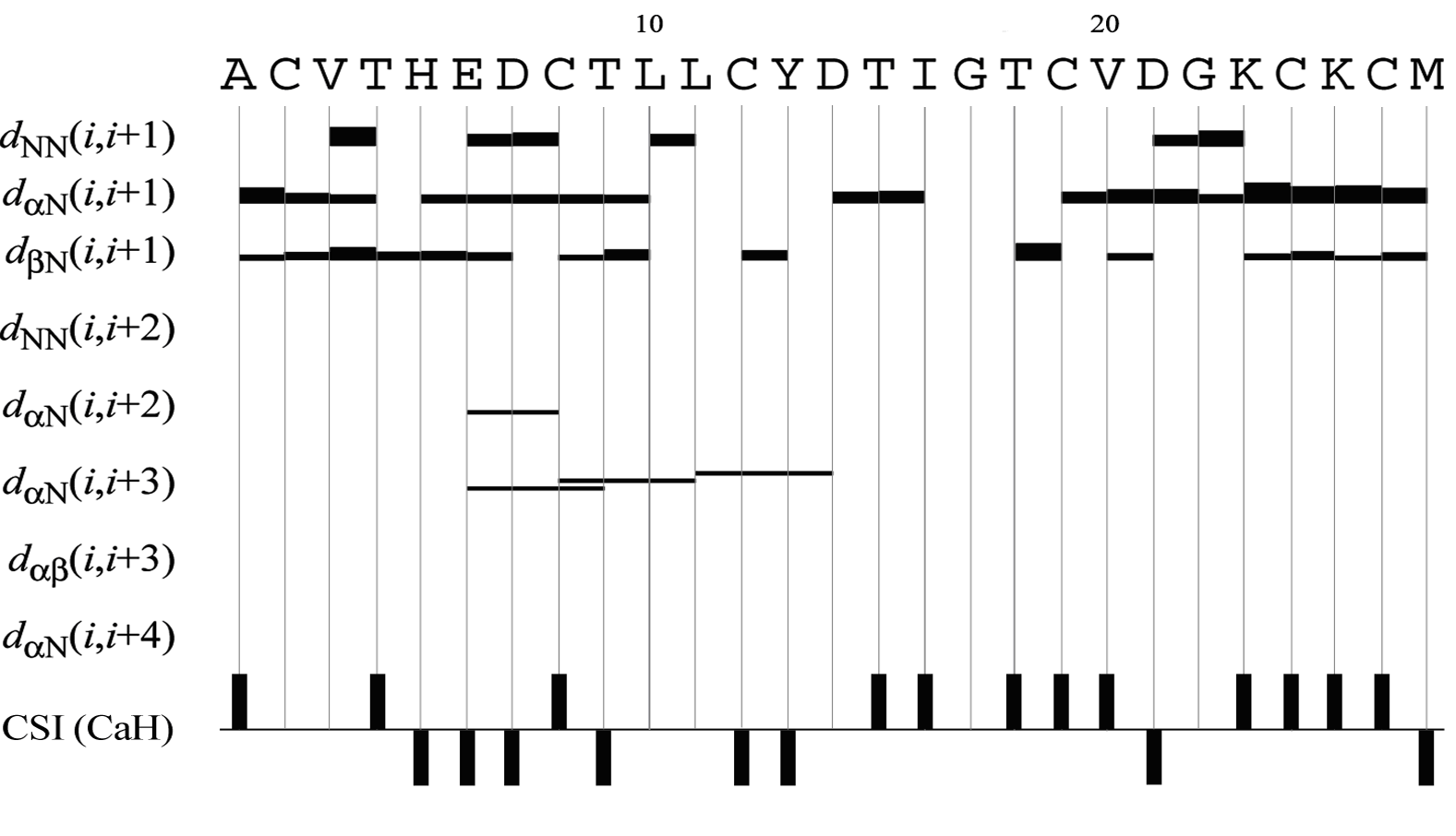

Supplement: Figure S4 — Summary of the NOE connectivities according to NOESY spectrum at 120 ms of mixing time and CαH chemical shift index (CSI). Bar thickness indicates the intensity of NOE connectivities, with thicker bars representing stronger NOEs. (TIF) [file pone.0035154.s004.tif]

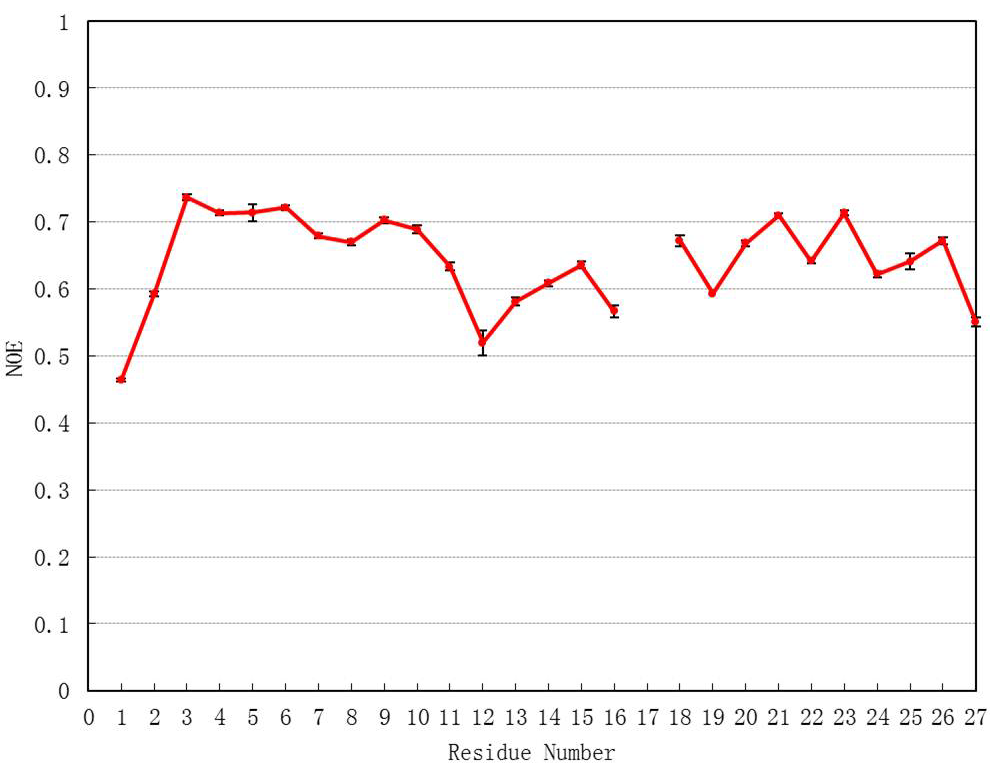

Supplement: Figure S5 — Values of backbone amide heteronuclear 1H–15N NOEs of ImKTx104. The NOE values are shown by red spot and line by residue number, and black bars indicate standard deviations. (TIF) [file pone.0035154.s005.tif]

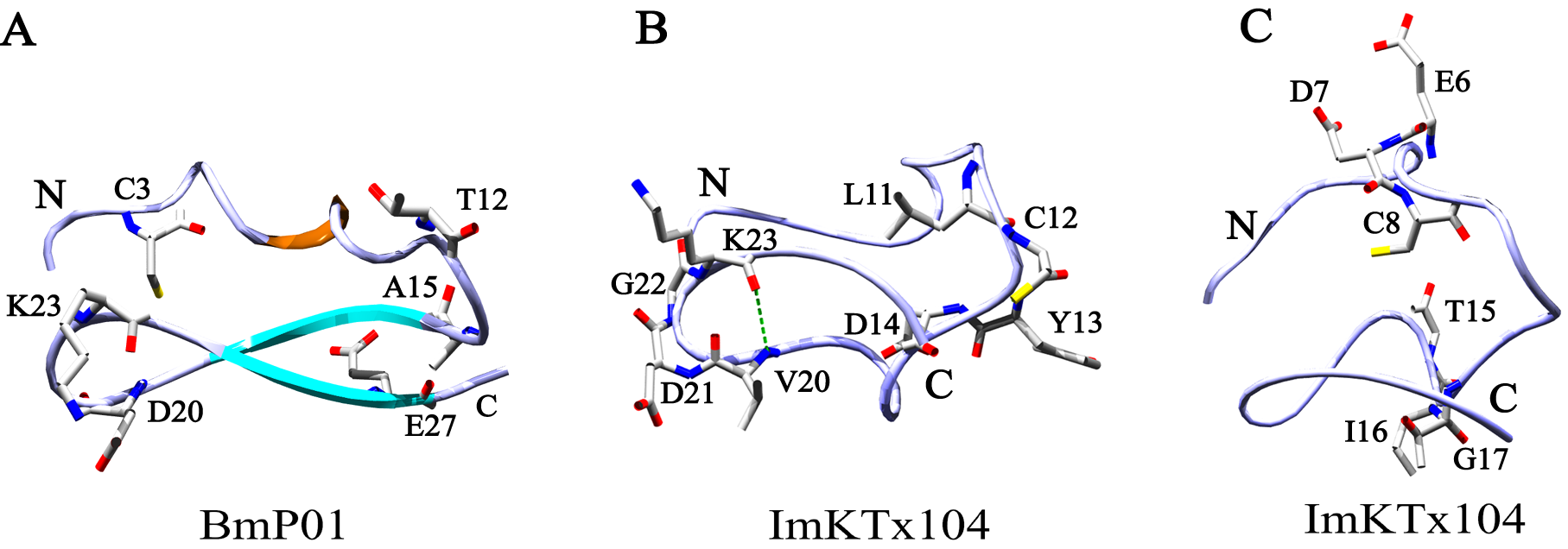

Supplement: Figure S6 — Structure comparison of ImKTx104 with the classical CSα/β scorpion toxin BmP01. (A) Structure of BmP01 (PDB code:1WM7). The representative amino acid residues were marked. (B) and (C), Structure of ImKTx104 (PDB code:2LIX). The representative amino acid residues were also marked. (TIF) [file pone.0035154.s006.tif]

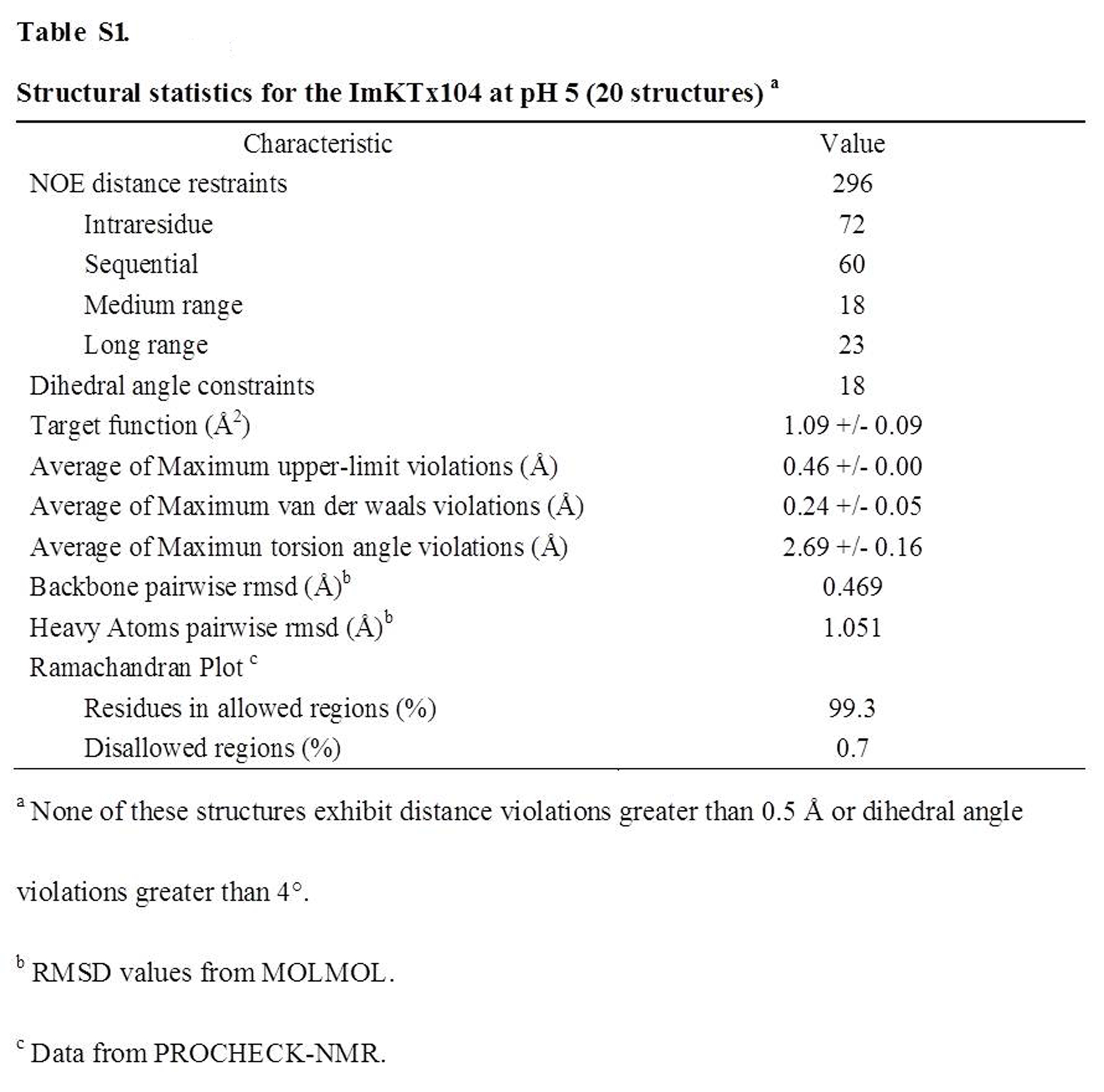

Supplement: Table S1 — Structure statistics for ImKTx104 at pH 5 (20 structures). (TIF) [file pone.0035154.s007.tif]
